# Boron Neutron Capture Therapy: From Nuclear Physics to Biomedicine

**DOI:** 10.3390/biology10050370

**Published:** 2021-04-26

**Authors:** Silva Bortolussi, Yuan-Hao Liu, Ignacio Porras

**Affiliations:** 1 Department of Physics, University of Pavia, 27100 Pavia, Italy; silva.bortolussi@unipv.it; 2 Unit of Pavia, National Institute of Nuclear Physics (INFN), 27100 Pavia, Italy; 3 Neuboron Medtech Ltd., Nanjing 211112, China; yhl.taiwan@gmail.com; 4 Department of Nuclear Science and Technology, Nanjing University of Aeronautics and Astronautics (NUAA), Nanjing 211106, China; 5 Department of Atomic, Molecular and Nuclear Physics, University of Granada, 18071 Granada, Spain

Boron Neutron Capture Therapy (BNCT) is a binary radiation treatment exploiting a nuclear reaction occurring in tumor cells. It consists of two steps: the first one is the administration of a borated drug to the patient, which is able to concentrate mostly in the tumor. The second one is irradiation with low-energy neutrons. The interaction of neutrons with biological tissue is complex, but the neutron capture with the stable 10-boron isotope (^10^B) occurs with high probability and produces two high-energy charged particles inside the cells. These particles (^4^He and ^7^Li nuclei) release all their energy in a path comparable with the cell dimension [1]. This nuclear property has the following advantages: 1. If boron concentration is sufficiently higher in the tumor than in normal tissues, more nuclear reaction will occur in the tumor with a differential dose deposition; 2. This dose is highly localized in tumor cells, allowing a substantial sparing of normal tissues. BNCT can be interpreted as a sort of internal hadrontherapy, where charged particles are generated directly inside the cells.

BNCT is the only radiotherapy whose selectivity is not based on the irradiation beam; rather, it depends on targeting boron carriers. This makes it possible to irradiate larger volumes of organs/tissues, hitting isolated tumor cells, possible sources of recurrence. For this reason, BNCT is a strong candidate to treat disseminated cancer, such as metastatic spreads, infiltrated tumors, or nodules critically close to very radiosensitive targets. BNCT can also be an adjuvant option, in combination with other pharmacological or radiation therapies.

BNCT has been applied to several hundreds of patients in different facilities in the world, where neutron beams were available from research nuclear reactors [2]. The needed neutron intensity was, in fact, only possible with this kind of installation in the past, able to ensure treatment times of the order of 1 h. However, in recent years, the technological innovation has led to the use of proton or deuteron accelerators coupled to Be, Li or C targets to obtain suitable neutron beams for clinical BNCT [3]. The easier authorization, use and maintenance of such machines has created a new BNCT era. In Japan, two BNCT facilities based on accelerators are currently treating patients and one is under clinical trial [4], while other installations are under commission in China, South Korea and Finland, and several others have been designed or are under construction.

The new BNCT era poses different challenges to reaching the goal of establishing a routine treatment for patients who suffer from non-treatable diseases. The search for innovative boron vectors, more efficient in selectively loading the tumor with sufficient ^10^B atoms, and in releasing the charged particles close to the cell nucleus, is very active. Together with the new neutron source, the availability of a novel borated formulation will represent a real breakthrough in BNCT effectiveness and applicability. However, there are many other fields of research which need to be deepened. The radiobiological BNCT effects, as a function of the absorbed dose, need to be understood for the different tumors: in vitro and in vivo experiments are urgently needed to produce new knowledge on BNCT effectiveness and potential. New dosimetric models are needed to express BNCT dose in photon-equivalent units towards a reliable in-patient dosimetry, able to allow predictions on the treatment outcomes based on the long-standing clinical experience with photon therapy. More interconnections between standard photon therapy and charged particle therapy methods and BNCT should be fostered to establish a common language that can be used to compare and eventually combine treatments. Novel methods to evaluate the therapeutic potential of neutron beams and analyze the safety for out-of-beam organs are necessary in view of the construction of new clinical facilities. More accurate experimental dosimetric techniques are also desired. Last but not least, more clinical results are important to establish reliable statistics and an indication of how to improve the tumor control, patient survival, and quality of life. All these topics and more are expected to be described in papers collected in this *Biology* Special Issue, offering a perspective on the future of BNCT’s clinical applications in biomedicine.

BNCT is an intrinsically multidisciplinary field of study. To create a new facility and transform it into a clinical center, different experts are involved: physicists, engineers, biologists, chemists, pharmacologists, and medical doctors must cooperate to complete all the necessary steps, from nuclear physics to biomedicine. In this sense, BNCT poses the challenge of real communication between different areas of science towards a common goal. As in all complex enterprises, only the sharing of knowledge and the union of different points of view can lead to real innovation. In this spirit, we welcome researchers involved in BNCT to submit their vision and their results, which are already part of this new BNCT era.

## Data Availability

Not applicable.
